# A pilot programme of community volunteers to improve hepatitis B medication adherence, Kiribati, 2025

**DOI:** 10.5365/wpsar.2026.17.3.1348

**Published:** 2026-07-15

**Affiliations:** a Department of Internal Medicine, Tungaru Central Hospital, Ministry of Health and Medical Services, Tarawa, Kiribati.; b Hepatitis B Free, Sydney, New South Wales, Australia.; c Departments of Internal Medicine and Pediatrics, Center for Space Medicine, Baylor College of Medicine, Houston, Texas, United States of America.

## Abstract

**Problem:**

Antiviral therapy for chronic hepatitis B (CHB) reduces morbidity and mortality and improves quality of life. Achieving high levels of adherence is challenging due to multiple barriers, including medication fatigue, stigma, transportation and limited health literacy.

**Context:**

Suboptimal adherence to antiviral therapy for CHB is problematic in Kiribati, particularly among high-risk groups including pregnant women and individuals with cirrhosis.

**Action:**

During January–August 2025, a community volunteer intervention was initiated on Kiritimati, a remote island of Kiribati, to address adherence to CHB antiviral therapy. Community volunteers were assigned to patients positive for hepatitis B surface antigen who were commencing antiviral therapy and were pregnant or had cirrhosis. During home visits, volunteers provided education and encouraged medication adherence and clinic follow-up. Adherence in the intervention group was compared with adherence in positive patients being treated on other remote islands without the intervention.

**Outcome:**

Adherence was measured by medication bottle counts. Outcomes were assessed by overall group adherence and individual adherence > 80%. Group adherence was 77.4% (240.5/310.75) in the volunteer intervention group compared with 52.5% (700/1333) in the group without it (*P* < 0.001). Overall adherence among pregnant women in the intervention group was 89.7% (52/58). Individual adherence > 80% was met in 58.8% (30/51) of the intervention group and 34.1% (70/205) in the control group (*P* = 0.0012).

**Discussion:**

Although findings are preliminary and the intervention and control groups were not matched, this pilot suggests community volunteers are a promising intervention to improve CHB treatment adherence in a high-prevalence Pacific island setting. This strategy could also be explored for other chronic conditions where adherence is important.

## PROBLEM

Chronic hepatitis B (CHB) is a significant global health concern affecting approximately 254 million people worldwide, (*1*) with sequelae of cirrhosis, liver failure and hepatocellular carcinoma (HCC). While effective antiviral medications are available to manage CHB, adherence to these treatments remains a significant challenge. Antiviral adherence is crucial for achieving and maintaining viral suppression and improving long-term outcomes and quality of life. Studies demonstrate that poor adherence to CHB medications can lead to treatment failure, viral resistance and increased risk of liver-related complications, including HCC. (*2*, *3*)

Multiple factors affect adherence to CHB antiviral therapy. The long-term nature of treatment, often lifelong, can lead to treatment fatigue and decreased motivation over time. (*4*) Absence of symptoms during early stages, lack of understanding about CHB and treatment, and side-effects (though usually mild) can also contribute to poor adherence. (*5*-*7*) The costs of treatment and transportation, family obligations and childcare, and stigma are additional factors. (*7*) Addressing social determinants of health is critical to improving adherence rates. The objective of this project was to assess the feasibility and effectiveness of an innovative, volunteer-driven medication adherence pilot programme in a remote setting.

## CONTEXT

Kiribati, a Pacific island nation with a high prevalence of hepatitis B (10–15%), implemented a national treatment programme in 2018. (*8*) Routine programme monitoring and interviews with health-care workers and patients identified medication adherence as a major challenge, particularly in critical populations such as patients with cirrhosis and pregnant women, in whom poor adherence can impact rates of HCC and vertical transmission. Based on clinic attendance rates, it was estimated that only 20% of all patients who started antivirals continued to take their medications as prescribed. Antiviral therapy is provided free of charge, but other factors such as family and caregiver responsibilities and limited health literacy are commonly cited by patients and local health providers as barriers to adherence. The cost of local transportation for patients to attend clinics and the need to maintain a consistent supply of medications across the vast geographical distances separating remote islands from the central pharmacy pose additional challenges. Systematic monitoring and implementation of strategies to improve adherence are essential in this resource-limited setting.

Addressing ongoing vertical transmission of hepatitis B is critical in Kiribati, given the persistently high prevalence of hepatitis B among children < 5 years old (3%). (*9*) In addition to supporting robust birth dose and infant vaccination coverage, integration with antenatal care for pregnant women who test positive for hepatitis B surface antigen (HBsAg) and antiviral therapy per World Health Organization guidelines are important strategies to address high rates of vertical transmission.

## ACTION

In January 2025, a community-level, health volunteer-driven pilot programme was initiated on Kiritimati, an outer island of Kiribati with a population of 7300. Individuals with CHB commencing antiviral therapy who were pregnant or diagnosed with cirrhosis (clinically or through transient elastography) were eligible. These groups were selected because prevention of vertical transmission and treatment of patients at high risk for progression are programme priorities.

The Ministry of Line and Phoenix Islands Development endorsed the project and its evaluation. Community volunteers (CVs) were recruited based on their interest in helping their community and proximity to patients in their villages. CVs received in-person training and ongoing telehealth support from a Kiribati physician. During a 1-day training session, CVs were instructed to provide basic education, to ensure that medications are taken and refilled, to counsel about side-effects and to facilitate clinic attendance. Emphasis was placed on accurately recording medication usage in a registry and developing positive relationships with patients. CVs transmitted monthly reports of their activities to the Kiribati physician. In addition to attending routine clinic appointments, pregnant women (*n* = 9) were visited weekly and patients with cirrhosis (*n* = 42) were visited monthly at home by volunteers. CV visits to pregnant women were scheduled more frequently because of the importance of adherence in preventing vertical transmission. CVs were paid 2 Australian dollars per visit. This programme began at the same time as an antiviral treatment programme on Kiritimati. All patients, whether assigned a CV or not, were naïve to antiviral therapy.

Patients were given 1 month (one bottle) of tenofovir alafenamide (TAF) at each clinic visit or, if unable to attend, by the CV during home visits. Adherence was calculated both by group and individually (**Box 1**). During home visits, CVs measured adherence through bottle counts.

Box 1 **Equations for treatment adherence calculations**

,

where *x taken* is in months (bottles) taken, and *y treatment time* is in months since treatment start.

.

Outcomes were group adherence, defined as the total number of bottles (months) consumed by the entire group divided by the total number of months that the individuals in the group had been on treatment. Individual adherence was defined as taking > 80% of the prescribed medications during the period that the individual was on treatment. Individual adherence in each group was defined as the proportion of individuals in the group who had > 80% adherence. Adherence was calculated on an intention-to-treat basis. Patients lost to follow-up were assumed to be non-adherent. For patients who died during the study period, adherence was based on the number of months on treatment until the month of death. For pregnant women, treatment time was calculated from the treatment start date until delivery.

At 6 months, an evaluation of programme effectiveness was undertaken to determine if the programme should be continued. Adherence (group and individual) in the volunteer intervention (VI) group was compared to that of CHB patients on treatment on other Kiribati outer islands (Abemama, Butaritari, Nonouti, Tabiteuea Maiaki, Tabuaeran and Teraina), where antiviral treatment had begun but the volunteer programme had yet to be implemented (NVI). Data from all patients from these islands whose adherence was assessed were used in the comparison. Adherence measurements were done through a patient registry of medication refills at outer island clinics. The total number of refills over the period since starting treatment was calculated for both the entire group and individuals (**Box 1**). A two-tailed *t*-test and a *Z*-test were used to analyse the differences in means and proportions, respectively. Differences were considered significant at *P* < 0.05. This comparison was not intended to be a formal clinical trial; rather, it is a progress report to inform policy-makers whether the programme is worthwhile to continue.

## OUTCOME

From January to August 2025, the 51 patients in the VI group were followed by CVs on Kiritimati Island. Of these, nine were pregnant women and 42 were patients with cirrhosis (23 male, 19 female). Of those with cirrhosis, four died and five were lost to follow-up. The total number of treatment months for the VI group was 310.8, and 240.5 bottles of TAF were consumed, giving an overall adherence rate of 77.4% (**Table 1**). Within this group, the pregnant patients had an overall adherence of 89.7% (52/58 treatment months; data not shown). During a similar time frame, the NVI group (*n* = 205) had a total of 1332 treatment months, with 700 bottles consumed, yielding a group adherence of 52.6%. The difference in means between the two groups was significant via a two-tailed *t*-test (*P* < 0.001).

**Table 1:** Characteristics and treatment adherence in volunteer and non-volunteer intervention groups of hepatitis B patients, Kiribati, 2025

Variable	Group		*P*
	Volunteer intervention	No volunteer intervention	
**Total no.**	**51**	**205**	
**No. (%) male**	**23 (45.1)**	**108 (52.7)**	
**No. (%) female**	**28 (54.9)**	**97 (47.3)**	
**Mean age (years)**	**39.4**	**37.8**	
**No. of months on treatment**	**310.8**	**1333.0**	
**No. of bottles consumed**	**240.5**	**700.0**	
**Group adherence (%)**	**77.4**	**52.5**	** < 0.001**
**Individual adherence (%)**	**58.8**	**34.1**	**0.0012**

Regarding individual adherence, 58.8% (30/51) of patients in the VI group maintained > 80% adherence, compared with 34.1% (70/205) in the NVI group (*P* = 0.0012). A high percentage (88.9%, 8/9) of the pregnant women in the VI group maintained adherence > 80%. Because pregnancy status was not recorded in the NVI group, individual adherence among pregnant patients could not be compared between the two groups.

In conversations with the Kiribati physician, patients and volunteers expressed enthusiasm about participating in the project. Health-care workers, often nurses or medical assistants, anecdotally commented on the utility of the programme, as it seemed to improve clinic attendance, reduce complications and enhance clinic efficiency.

## Discussion

This pilot intervention suggests that CV support improves adherence to CHB antiviral therapy among high-priority groups of pregnant women and patients with cirrhosis living in a remote, low-resource setting on Kiritimati, Kiribati. CVs can potentially address several reasons for non-adherence, such as side-effects, misinformation, transportation issues, medication fatigue and stigma. Patient acceptance of the programme was enhanced by recruiting volunteers living in the same community who understand the challenges local patients face. To help patients with difficulties attending clinic, some CVs delivered medication to patients’ homes. Pregnant patients had adherence rates approaching 90%. This may be attributed to more frequent visits (weekly) by the CVs and additional opportunities for education. Although a quantitative economic analysis was not performed, the project cost (roughly 100 Australian dollars per month) was small compared with the total hepatitis programme budget and the cost of hospitalizations due to CHB complications. The intervention costs could easily be recouped by preventing one maternally transmitted case of hepatitis with its associated lifelong health consequences. Improvements in adherence may have a significant impact on CHB mortality. A modelling study published in 2016 predicted that an increase in adherence from 65% to 95% would result in a 27% decrease in worldwide CHB mortality over 15 years. (*10*) Preventing new infections and the worsening of existing infections can avoid significant health costs caused by hepatitis-related morbidity and mortality.

There is limited research on strategies to improve adherence to CHB antiviral therapy in low- and middle-income countries (LMICs), including the impact of CVs. (*11*) Most data originate from HIV- and HCV-infected patients, and adherence is not a measured outcome. Li et al. performed a meta-analysis examining the role of community engagement and public health interventions on hepatitis outcomes. (*12*) They reported improvement of such programmes on HBV/HCV treatment adherence (relative risk: 1.14, 95% confidence interval: 1.03–1.27); however, data are sparse and mostly from high-income countries. Downs et al. described the efficacy of a peer support programme and its positive effects. (*13*) Other research in LMICs demonstrated that community supporters improved adherence to HIV antiretroviral therapy, especially in combination with text messaging. (*14*) Although more evidence is needed, these studies suggest the potential value of community-based interventions.

Our pilot project has several limitations. It was non-randomized, with a small sample and short study period. Because the pilot programme began concurrently with the initiation of antiviral treatment on Kiritimati, we did not have historical data to compare adherence in the VI group pre- and post-intervention. While the VI group included only pregnant women and people with cirrhosis, the NVI group included pregnant women and patients with and without cirrhosis. Patients without cirrhosis are likely to have fewer symptoms and are possibly less motivated to maintain adherence. Pregnant women may be more adherent to prevent transmission to their baby or less motivated if they have safety concerns about the medications. We were unable to make a direct comparison of adherence among pregnant women between the two groups because the duration of antiviral therapy for pregnant women in the NVI group was not recorded in the registry. Differences in local factors on other outer islands without the intervention programme may also introduce bias. For example, access to health facilities varies widely across the many islands included in the study. While they share a common language, the VI group is located in the Line Islands, thousands of kilometres from the Gilbert Islands where the NVI group resides, and some variations in regional customs and beliefs may exist. There is no gold standard measurement for CHB antiviral therapy adherence. We adopted a simple measure based on bottle counts, which is less precise than directly observed therapy or individual pill counts. To address these limitations, we plan to conduct a randomized trial over 12 months with larger, matched cohorts that are enrolled simultaneously. Future economic analysis may also help to quantify the economic benefits of such a programme. These studies may help guide policy-makers in developing strategies to improve adherence.

Our findings should be taken as preliminary and hypothesis-generating, rather than definitive evidence of effectiveness. Despite their limitations, the results demonstrate a promising innovation that may ameliorate barriers to CHB antiviral adherence in a low-resource setting. Strong telehealth support and communication among the lead Kiribati physician, CVs and mid-level clinicians who provide most of the hepatitis care on the outer islands enabled continual feedback and rapid data-sharing. However, larger-scale implementation will require more resources. Our findings can potentially be generalized to other chronic diseases. A scoping review by Hassan et al. studied the effectiveness of community-based interventions for the management of chronic conditions in LMICs. (*15*) Facilitators influencing successful implementation outcomes included strong motivation among community health workers, high-quality training and role recognition for community health workers. Additionally, the presence of CVs from local neighbourhoods promoted positive interpersonal relationships, and assigning female CVs to pregnant patients produced positive gender dynamics.

Improved adherence can reduce vertical transmission, decrease hospitalizations, and lessen complications, including HCC and deaths. CHB patients commonly have comorbidities requiring medications, which could also be addressed during CV visits. Adherence is essential for other communicable and noncommunicable diseases, such as diabetes, hypertension and heart disease. Fostering a supportive health-care environment in the local community that reduces stigma, promotes education and encourages open communication can empower patients to adhere to their treatment.
